# Nanoindentation of Graphene-Reinforced Silica Aerogel: A Molecular Dynamics Study

**DOI:** 10.3390/molecules24071336

**Published:** 2019-04-04

**Authors:** Sandeep P. Patil

**Affiliations:** Institute of General Mechanics, RWTH Aachen University, Templergraben 64, 52062 Aachen, Germany; patil@iam.rwth-aachen.de; Tel.: +49-241-80-90036

**Keywords:** silica aerogel nanocomposites, nanoindentation, molecular dynamics

## Abstract

In the present work, we performed nanoindentation tests using molecular dynamics (MD) simulations on graphene, native silica aerogels, and single- and multi-layered graphene-reinforced silica aerogel nanocomposites. This work mainly focused on the two aspects of nanoindentation simulations: first, the resultant indentation force–depth curves, and second, the associated mechanical deformation behavior. We found that in the single-layer graphene-reinforced silica aerogel nanocomposite, the indentation resistance was four-fold that of native silica aerogels. Moreover, the combined system proved to be higher in stiffness compared to the individual material. Furthermore, the indentation resistance was increased significantly as we proceeded from single- to two-layered graphene-reinforced silica aerogel nanocomposites. The results of the study provide a detailed understanding of the mechanical behavior during the indentation tests of nanocomposites, which helps to design advanced nanoscale multi-layered materials.

## 1. Introduction

The structure of silica aerogel is a three-dimensional open-cell nanoporous network whose skeleton is composed of interconnected silica nanoparticles [1]. Due to the nanoporous structure, it has extraordinary physical properties such as extremely low thermal conductivity (∼0.01 W/mK), low sound speed (∼100 m/s), low refractive index (∼1.05), and dielectric constant (1.0–2.0) [2,3]. Hence, silica aerogel has been used as a heat and sound-insulating material in buildings and chemical plants, as a super-capacitor for energy storage, as a catalyst carrier for chemical reactions, as a Cerenkov counter in high-energy physics, and as a highly-energetic particle collector in aerospace [1,4,5]. However, what blocks the further applications of silica aerogels is their brittle nature. Moreover, due to the high porosity in combination with a low fraction of fully-connected masses of silica nanoparticles, silica aerogels exhibit low tensile strength.

Therefore, several approaches have been proposed in the literature to improve the mechanical properties of silica aerogel, e.g., chemically modifying the aerogels via integration of organic coatings, organic crosslinking of the backbone or using organically-modified silica precursors [6,7]. However, this approach compromises some of the advantages of the inorganic aerogels, such as their zero volatile organic compound release and zero flammability, or the required aerogel densities are quite higher for certain applications. A prominent approach to improve the mechanical properties of silica aerogel is by reinforcement. Many researchers have focused on silica aerogel composites by the addition of particles, polymers, or fibers. Recently, carbon nanomaterials such as carbon nanofibers [8,9], carbon nanotubes [10,11], carbon aerogels, and graphene [12,13,14], are being used as reinforcement materials in silica aerogels. However, to the best of our knowledge, surprisingly, these composite materials have never been investigated before using molecular dynamics (MD) simulations.

Computational modeling has not been used extensively to explore many aspects of silica aerogels. However, in the last decade, there has been a rising interest in MD simulations, which have been used to describe the mechanical properties of native silica aerogels [15,16,17,18,19,20,21,22]. Due to the real-time scales (picoseconds), it is a powerful technique to investigate the mechanical properties, interface mechanics, and crack dynamics at an atomistic scale [20,22,23]. In our previous work, large-scale MD simulations were used to study the inelastic effects under large deformations for a wide range of silica aerogel densities [20]. Moreover, we examined the crack propagation and the crack length effect on the resulting fracture properties, such as fracture strength, fracture toughness, and strain energy release rate [22].

In the past three decades, nanoindentation experiments have been predominantly used to study the elastic and inelastic behavior of several porous materials. In particular, these experiments allow the measurement of physical properties such as Young’s modulus, hardness, and the elastic properties of heterogeneous nanomaterials with high spatial resolution (nanoscale) [24]. In the literature, the mechanical properties of silica aerogels were evaluated in several studies of nanoindentation experiments [25,26,27,28,29,30]. In our recent work, we performed nanoindentation tests via MD simulations on native silica aerogels using a spherical indenter to investigate the mechanical properties and the deformation behavior [23]. For a wide range of densities of silica aerogels (274–847 kg m${}^{-3}$), the experimentally-measured elastic moduli are in good agreement with MD simulation results. Moreover, MD simulations provided a detailed understanding of the nanomechanics during the nanoindentation on low-density silica aerogels [23].

In this work, we aim to elucidate the mechanical properties of graphene, native silica aerogels, and graphene-reinforced silica aerogel nanocomposites during nanoindentation tests using MD simulations. In particular, we focused on indentation force–depth curves to compare the influence of graphene reinforcement on silica aerogel.

## 2. Methods and Materials

In this work, all MD simulations of the nanoindentation were performed using the Large-scale Atomic/Molecular Massively Parallel Simulator (LAMMPS) [31]. For visualization and analysis of the atomistic simulation data, we used OVITO [32]. The interatomic interaction of silica was modeled using the Vashishta potential [33,34], which takes into account not only the pairwise interactions, but also considers the energy associated with the bonding angle and the orientation of three atoms.

### 2.1. Creation of the All-Atom Silica Aerogel Model

A detailed description of the creation of the atomistic silica aerogel models has been discussed in our previous work [20]. Here, the building of an all-atom model of silica aerogel is briefly described. The silica aerogel model was built starting with β-cristobalite atomic coordinates [35]. Initially, to generate bulk silica, the periodic boundary conditions were assigned in three mutually-perpendicular directions. The velocity-Verlet algorithm was used to solve the equations of motion of the particles with a time step size of 0.5 fs. A random velocity was assigned to all atoms at 7000 K. Subsequently, the sample was quenched at constant volume and a constant number of atoms to 300 K at 5 K/ps, which was followed by a system energy minimization using the conjugate gradient method. Finally, amorphous silica was generated by the sample relaxation at atmospheric conditions (300 K and 1 bar). The relaxation of the amorphous silica sample was followed by an instantaneous triaxial expansion to the desired density ρ = 403 kg m${}^{-3}$.

The expanded sample was then heated to 3000 K for 50 ps, where the MD system was given the opportunity to overcome energetic barriers in the search for conformations with energies, followed by relaxation. Finally, the sample was quenched to 0 K followed by the energy minimization. The nanoporous silica aerogel was formed when the sample was further brought back to the atmospheric conditions. The heat treatments in the simulations were performed using the NVT (constant volume and constant temperature) ensemble except for the relaxation, which was carried out using the NPT (isothermal–isobaric) ensemble. The nanoindentation tests were carried out on the equilibrated systems.

The separated distance between two atoms was computed using the radial distribution function (RDF) or g(r). Accordingly, the pair distances for Si–Si, Si–O, and O–O were calculated using the first RDF peak as 3.066 Å, 1.609 Å, and 2.626 Å, respectively. These pair distances show good agreement with the literature [16,20,33]. A detailed discussion of the RDF is included in the Supplementary Materials (see Figure S1).

### 2.2. Simulation Details

In this work, we mainly focused on the deformation mechanisms of highly-nanoporous silica aerogels and nanocomposites of them under indentation at the nanoscale. For the reinforcement, we used graphene sheets in silica aerogels. For MD simulations of graphene, the short-range interactions between the C–C atoms was defined by Brenner’s second-generation reactive empirical bond-order (REBO) force field [36], while the van der Waals force (or Lennard–Jones potential) was adopted to describe the long-range interactions between the carbon atoms. Here, we used 2 Å as a truncated cut-off value for graphene fracture, which has been discussed in detail in previous studies [37,38,39].

**Figure 1:**
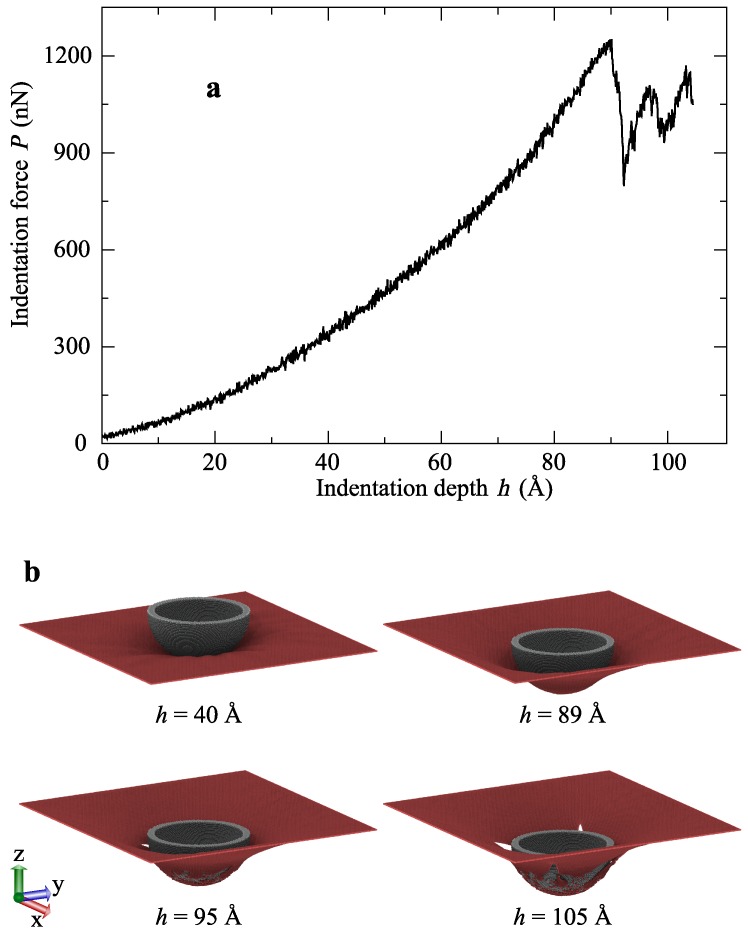
(**a**) Indentation force–depth (*P* – *h*) curve of the nanoindentation test of a graphene sheet. (**b**) Snapshots of the graphene-indenter system during indentation. The spherical indenter (gray) was moved downwards to fracture the graphene sheet (red). The first two snapshots show the deformation of the graphene before fracture, and the last two show the behavior of graphene after fracture.

Figure 1b shows snapshots of the graphene-indenter system during indentation, which consists of a spherical diamond indenter. In all the nanoindentation simulations, the diamond indenter contained carbon atoms and was assumed to be perfectly rigid. A spherical diamond indenter with a diameter of 160 Å was used, and the indentation was carried out with a constant linear velocity *v* of 1 Å/ps in the z-axis direction.

In the graphene-reinforced nanocomposite simulation systems, graphene sheets were modeled on and in between the silica aerogels. Therefore, it was crucial to define the interatomic potential between Si, O, and C atoms. As discussed earlier, for the silica atoms [33,34] and the C–C atoms [36], interactions have been defined with well-known interatomic potentials. However, the Si–C and O–C interactions remained to be defined in the simulations. For these interactions, we used the parameters proposed by Rappe et al. [40], which were based primarily on the short-range van der Waals potential [41]. The parameters ϵ and σ were set as 8.909 meV and 3.326 Å, respectively, for Si–C interaction, and 3.442 meV and 3.001 Å, respectively, for O–C interaction [42]. As the diamond indenter was treated as rigid in the simulation, the interactions within the indenter were ignored.

After the whole indentation system had been set up, the Maxwell–Boltzmann distribution was used to assigned the initial velocities and then followed by energy minimization [43]. Subsequently, every MD simulation system was equilibrated at atmospheric conditions for 100 ps using the NVT ensemble to reach a steady state. Finally, the indentation simulations were carried out with the NVE (constant volume constant internal energy) ensemble. In this work, various indentation models were considered, e.g., one-layer graphene-reinforced (1G-SiO${}_{2}$), two-layered graphene-reinforced with a single silica slab of Type I (2G-1SL-I-SiO${}_{2}$), three-layered graphene-reinforced with a double silica slab of Type I (3G-2SL-I-SiO${}_{2}$), two-layered graphene-reinforced with a single silica slab of Type II (2G-1SL-II-SiO${}_{2}$), and three-layered graphene-reinforced with a double silica slab of Type II (3G-2SL-II-SiO${}_{2}$). A description of all the indentation simulation models is listed in Table 1. Silica aerogels are highly nanoporous solids; therefore, indentation location plays an important role, e.g., the pore or the backbone is just beneath the indenter. Therefore, to avoid the location-dependent mechanical properties, six simulations were performed at different locations for every analysis, and the resultant average data are presented.

## 3. Results and Discussion

The primary goal of this study is to investigate the influence of graphene reinforcement on the mechanical properties of silica aerogel using nanoindentation. MD simulations of native silica aerogel and single and multi-layered graphene systems were carried out. Moreover, in multi-layered graphene-reinforced systems, two types of silica aerogel slabs were considered. Finally, the results of all the MD simulations are presented and compared.

### 3.1. Indentation of Graphene

Graphene is the highest stiffness and strength material known to man [44]; therefore, it is a promising candidate to act as a reinforcing filler in many materials. Thus, we first studied the load-carrying capacity of graphene using a spherical indenter. Initially, the distance between the indenter tip and the top surface of the graphene sheet was 15 Å. After the ideal travel of the indenter, in the progressive indentation, the resisting force on the indenter was offered from graphene, which was calculated at each simulation step and also averaged for 0.1 ps to avoid high fluctuations. In these graphene simulations, four-sided edge-atoms of the graphene were kept fixed during the indentation process, and the rest of the atoms were held at a constant temperature using the Langevin thermostat [45] as the thermostat atoms. The indentation was continued until the graphene sheet was fractured. During this process, indentation depth (*h*) and the associated indenter force (*P*) were recorded, and the averaged curve is shown in Figure 1a.

Figure 1b shows the deformation and the fracture behavior of the graphene sheet. After the cracks initiated (*h*> 90.1 Å), the fractured portion of carbon atoms tried to make connections with an unfractured graphene sheet. Therefore, even after the fracture, there was a significant amount of resistance offered from the sheet. In the indentation simulations of the graphene sheet, the fracture occurred in the graphene sheet at a depth of 90.1 Å, and the maximum force was reached at 1229 nN.

### 3.2. Indentation of Silica Aerogel

The distance between the indenter tip and the top surface of the native silica aerogel of density ρ = 403 kg m${}^{-3}$ was 15 Å. Therefore, the indentation loading was divided into three phases, ideal travel (15 Å downward), progressive indentation (90 Å downward), and unloading applied to the indenter (90 Å upward). To avoid the rigid-body movement of silica aerogels, the bottom portion of 30 Å was fixed during the nanoindentation. In this work, all the MD simulations were carried out using the displacement controlled loading, and the maximum penetration depth was fixed at 90 Å. The details of the indentation tests for a wide range of densities of native silica aerogel have been discussed in our recent work [23].

**Figure 2:**
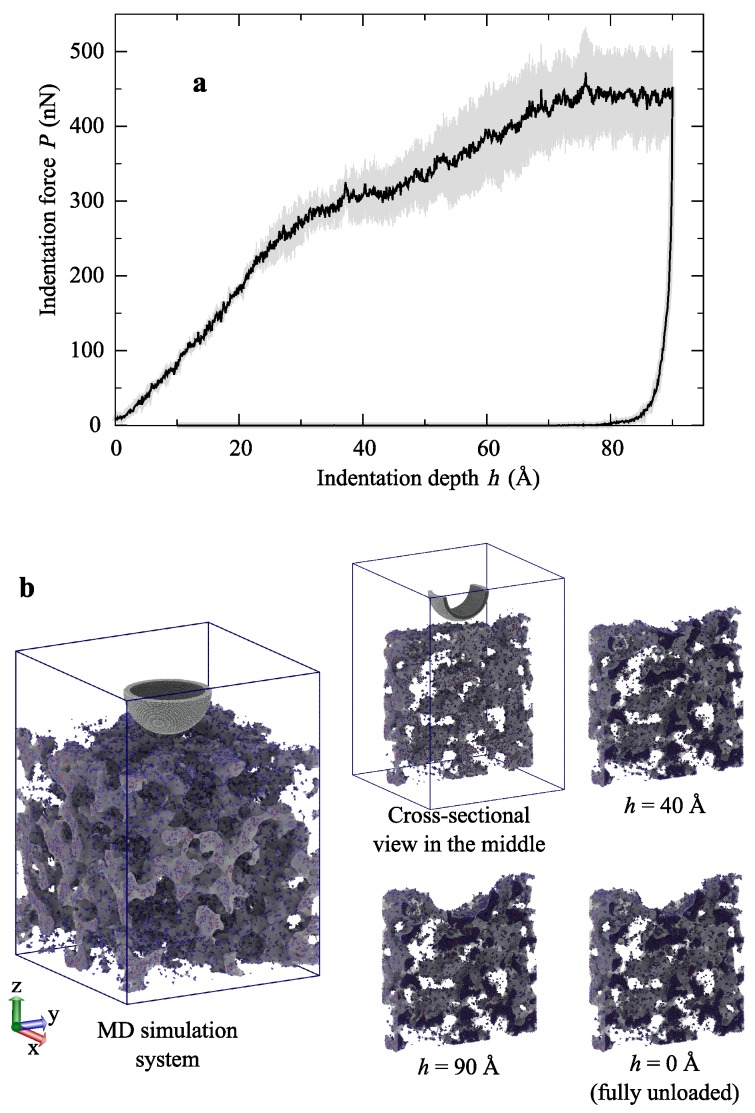
(**a**) Indentation force versus depth curve of the nanoindentation test on native silica aerogel. The black line is the average from indentation force–depth curves of different starting structure models, and the gray shaded area indicates the standard error deviation. (**b**) Snapshots of the native silica aerogel during the nanoindentation test. For understanding purposes, the deformation images of loading and unloading were taken at the middle cross-sectional view with a thickness of 80 Å.

Figure 2a shows the averaged *P* – *h* curve with the standard error deviation. To avoid the model-dependent *P* – *h* curve, independently, nanoindentation tests were performed on six faces of the silica aerogel block. For the maximum penetration of 90 Å, the maximum recorded force was 443 ± 58 nN. From the nature of the curve, it can be observed that the MD models showed a high plastic energy dissipation, which was nearly 97–98%. Therefore, there was nearly no recovery observed, which is demonstrated in Figure 2b. Until the point of the maximum depth was reached, the diamond-indenter made impressions in the aerogel. However, upon fully unloading the deformation, one would observe the permanent deformation in the native silica aerogel.

### 3.3. Single-Layer Graphene-Reinforced Silica Aerogel

**Figure 3:**
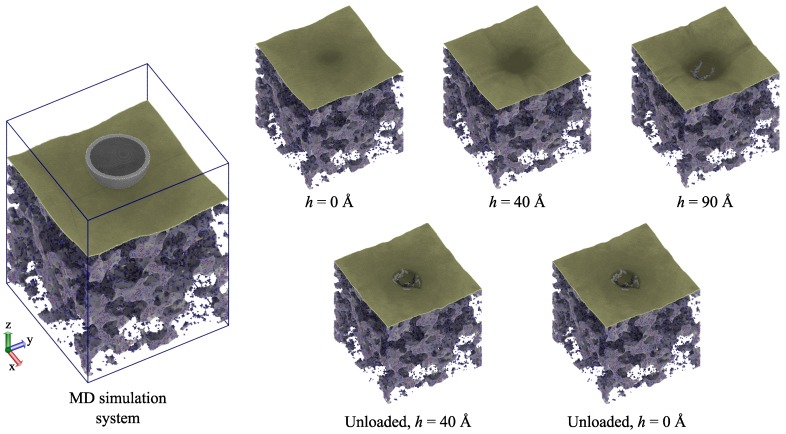
Nanoindentation on a 1G-SiO${}_{2}$ system. The snapshots depict the deformation of the graphene sheet and silica aerogel during loading and unloading.

To investigate the impact of graphene reinforcement on the mechanical properties, the MD model was constructed with a single layer of a graphene sheet on top of the native silica aerogel (1G-SiO${}_{2}$), as shown in Figure 3. Initially, the graphene sheet was modeled at a distance of 3 Å from the top of the aerogel block, which was placed there without any constraint on it. An annealing treatment was carried out to give the system an opportunity for the best conformations with energies. To this end, the system was heated from 300 K–800 K and then cooled down at 5 K/ps back to room temperature.

During loading, the graphene sheet transferred the indentation load to the aerogel. In the case of a single graphene sheet, the fracture occurred at *h* = 90.1 Å. However, for a 1G-SiO${}_{2}$ system, the fracture was observed before the *h* reached 90 Å. This occurred due to the out-of-plane flexibility of the graphene sheet, which was restricted by the deformed aerogel. Upon unloading, the permanent deformation of the fractured graphene sheet and the silica aerogel was observed.

**Figure 4:**
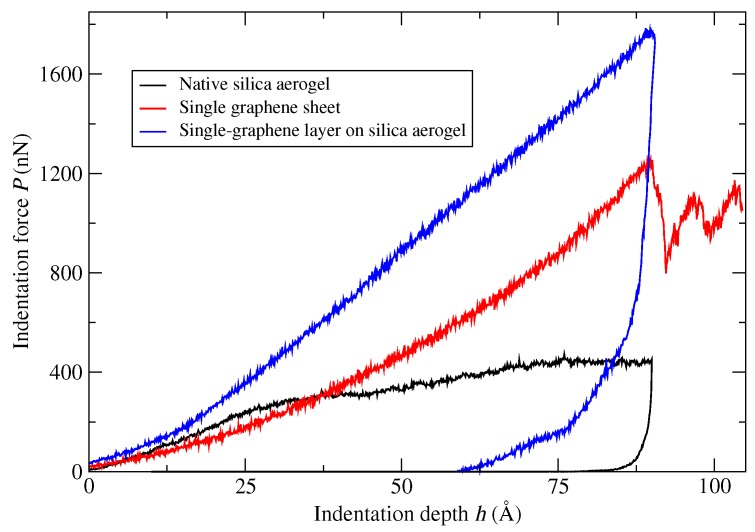
Comparison of indentation force versus depth curves of native silica aerogel, graphene sheet, and a 1G-SiO${}_{2}$ system.

Figure 4 compares the *P* – *h* relation of graphene-reinforced silica aerogel with the response of the single graphene sheet and that of native silica aerogel. The averaged maximum indentation forces for the graphene and the native silica aerogel were 1229 and 443 nN, respectively. For the 1G-SiO${}_{2}$ model, the maximum indentation force was 1771 nN, which was a four-fold increase in the maximum indentation force of native silica aerogel. Noteworthy was that the resistance force of graphene had also increased by 44%. Moreover, the graphene-reinforced silica aerogel model was much stiffer than the graphene, as well as the native silica aerogel. In the deformation of the graphene-reinforced silica aerogel, two important phenomena took place. First, the graphene sheet had to deform, and second, due to interactions between the graphene and aerogel, the concentrated force exerted by the indenter was distributed on a considerably larger area. As a result, the combined system offered high resistance against the deformation, which showed ultimately higher stiffness, as well as high maximum indentation force.

### 3.4. Graphene-Reinforced Multilayered Silica Aerogel Nanocomposites

To study the mechanical performance of the nanocomposites, mainly two thicknesses of slabs of the silica aerogel were modeled. To investigate the influence of multi-layered reinforcement accurately, the native silica aerogel model was kept at the base, and additional layers and slabs continued to be added. The Type I and II silica aerogel slabs represent the different thicknesses of 50.4 and 126.3 Å, respectively. Each type was constructed with two layers (2G-1SL-SiO${}_{2}$) and three layers (3G-2SL-SiO${}_{2}$) of graphene reinforcement. The details of the graphene-reinforced multilayered silica aerogel models are presented in Table 1. As discussed previously, the annealing heat treatment was carried out on all the simulation systems before the indentation tests.

**Figure 5:**
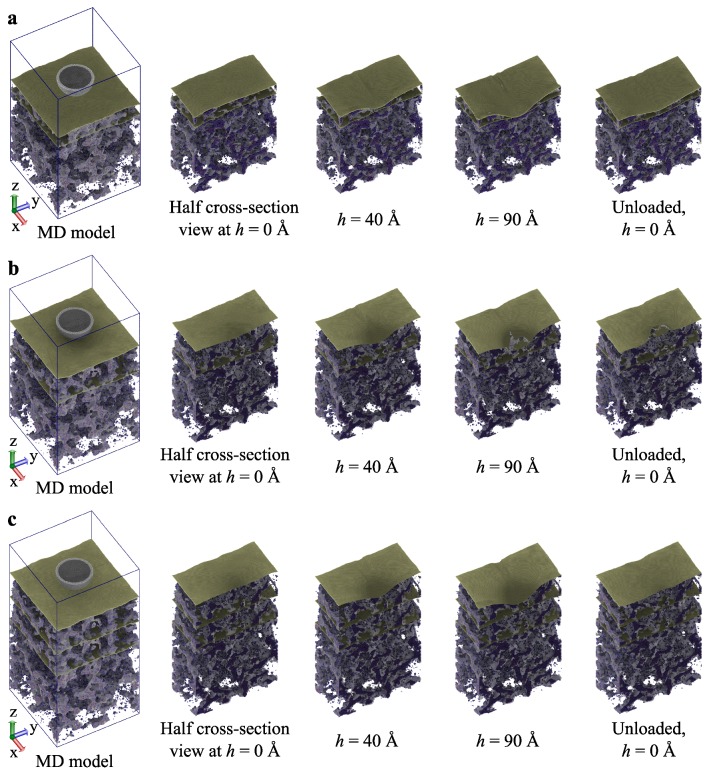
Deformation behavior of a two-layered (**a**) slab Type I, and a two-layered (**b**) and a three-layered (**c**) slab Type II graphene-reinforced silica aerogel nanocomposite observed during nanoindentation simulations.

Figure 5 represents the deformation of the multi-layered graphene-reinforced silica aerogel nanocomposite during nanoindentation simulations. In the case of simulations of the 2G-1SL-I-SiO${}_{2}$ model, due to the applied indentation force to the maximum depth of 90 Å, first the top graphene sheet and the aerogel slab of the thickness of 50.4 Å were deformed, and subsequently, the bottom graphene sheet was also deformed (see Figure 5a). There was no fracture in the graphene sheets observed. Moreover, after fully unloading, the graphene sheets nearly achieved their original shape and size. For the simulations of the 2G-1SL-II-SiO${}_{2}$ model, the fracture of the graphene sheet occurred (see Figure 5b). Here, the localized deformation around the indenter was perceived. After the unloading, the permanent deformation of the fractured graphene sheet and the silica aerogel was observed. Figure 5c shows the deformation simulation of the 3G-2SL-II-SiO${}_{2}$ model. As indentation progressed, the deformation of the graphene sheet, as well as the silica aerogel occurred, however without fracture of the graphene sheet.

In all simulations, as indentation progressed, the elastic deformation of the graphene sheets was observed; however, close to the indented area, below the graphene sheets, localized compression of silica aerogel was noticed. Although the interactions between the graphene and aerogel distributed the concentrated force exerted by the indenter on a considerably larger area, the localized compression of silica aerogel was significant. This localized area turned into local densification of the silica aerogel, which restricted the further elastic deformation of the graphene sheets and ultimately resulted in a fracture of the sheets. Interestingly, in the case of 2G-1SL-I-SiO${}_{2}$ composites, the thickness of the slab was much smaller compared to the maximum depth. Therefore, the indenter first started compressing the slab, and as the force continued, there was no possibility of further densification of the slab. Hence, it was easy for the force to deform the bottom graphene sheet without fracturing both. Moreover, as the number of graphene layers increased, the plastic deformation energy decreased significantly (see the detailed description in the Supplementary Materials).

**Figure 6:**
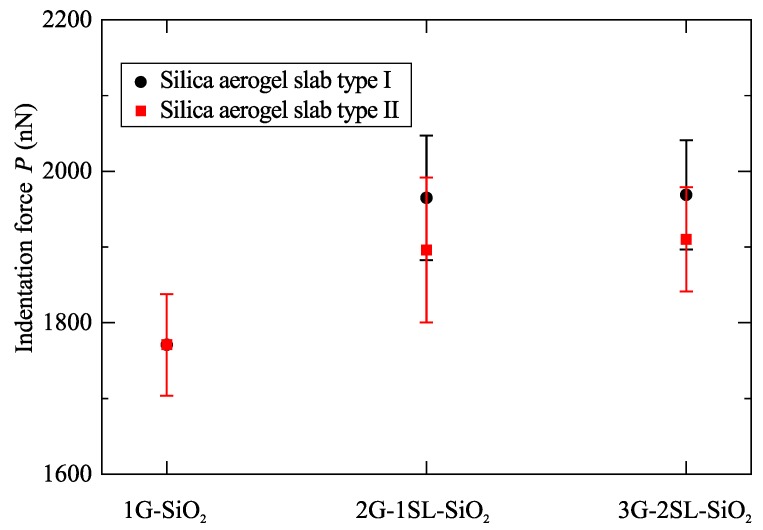
The maximum indentation force of the multi-layered graphene-reinforced silica aerogel systems when indented up to 90 Å.

Figure 6 shows the maximum indentation force of the multi-layered graphene-reinforced silica aerogel systems. As the number of graphene-reinforced layers increased with the additional silica aerogel slabs, the maximum indentation force also increased. This increase was significant from single- to two-layered graphene-reinforced silica aerogels; however, after two layers, the maximum indentation force was nearly constant. Furthermore, the different thicknesses of silica aerogel slabs had no significant influence on the maximum indentation force.

## 4. Conclusions

In this work, we have investigated the mechanical properties of a single graphene sheet, native silica aerogel, and multi-layered graphene-reinforced silica aerogel nanocomposites using MD simulations of nanoindentation tests.

Graphene has proven to be an extraordinary reinforcing candidate for silica aerogels on the basis of the indentation force–depth predictions. For the single-layer graphene-reinforced silica aerogel nanocomposite, the indentation resistance was four-fold of the indentation resistance of a native silica aerogel. The nanocomposite system also showed a higher stiffness compared to the individual material. The maximum indentation force was increased significantly as we proceeded from a single- to two-layered graphene-reinforced silica aerogel nanocomposite. Moreover, the different thicknesses of silica aerogel slabs had no significant influence on the maximum indentation force.

We studied here the multi-layered graphene reinforcement; therefore, the extension of this work is to distribute the graphene sheets randomly in silica aerogels and examine the influence of reinforcement. Furthermore, the popular reinforcement materials in silica aerogel, such as carbon nanofibers, carbon nanotubes, carbon aerogels, and graphene aerogels, will be studied using nanoindentation.

## Figures and Tables

**Table 1:** Description of the MD simulation models. G, graphene; SL, silica.

Simulation System	Model Size	Number of Atoms (millions)		
	x × y × z (Å${}^{3}$)	Graphene	Silica Aerogel	MD * Model
Graphene	∼407.0 × 407.0 × 3.4	0.127	-	0.127
Native silica aerogel	∼407.0 × 407.0 × 407.0	-	0.786	0.786
1G-SiO${}_{2}$	∼407.0 × 407.0 × 410.4	0.127	0.786	0.913
2G-1SL-I-SiO${}_{2}$	∼407.0 × 407.0 × 467.9	0.254	0.884	1.138
3G-2SL-I-SiO${}_{2}$	∼407.0 × 407.0 × 524.3	0.381	0.982	1.363
2G-1SL-II-SiO${}_{2}$	∼407.0 × 407.0 × 543.8	0.254	1.032	1.286
3G-2SL-II-SiO${}_{2}$	∼407.0 × 407.0 × 676.0	0.381	1.278	1.659

* For all the MD models, a half-spherical diamond indenter of a diameter of 160 Å with ∼62,400 atoms was used.

## Supplementary Materials

The following are available online at https://www.mdpi.com/1420-3049/24/7/1336/s1.
