# Supplementary material for: Nanoindentation of Graphene-Reinforced Silica Aerogel: A Molecular Dynamics Study
Source: Molecules. 2019 Apr 4;24(7):1336. doi: 10.3390/molecules24071336 (PMC6480658; doi:10.3390/molecules24071336)
Supplement: Supplementary file 1 [file molecules-24-01336-s001.pdf]

Supplementary Materials

Nanoindentation of graphene-reinforced silica aerogel: A molecular dynamics study

Sandeep P. Patil*

Institute of General Mechanics, RWTH Aachen University, Templergraben 64, 52062 Aachen, Germany
E-mail: patil@iam.rwth-aachen.de

Radial distribution function

The separated distance between the two atoms can be computed using the radial distribution function (RDF) or $g(r)$. The quantity r, which corresponds to the maximum value of $g(r)$, gives the pairwise distances between the respective atoms. In the present work, for Si–Si, Si–O and O–O the pair distances were calculated as 3.066 Å, 1.609 Å and 2.626 Å, respectively. These parameters were well within the range provided by Murillo et al. [1] and also validates the experimental results for bond length parameters for silica [2]: 1.61 ± 0.05 Å for Si–O, 2.632 ± 0.089 Å for O–O, and 3.08 ± 0.10 Å for Si–Si.

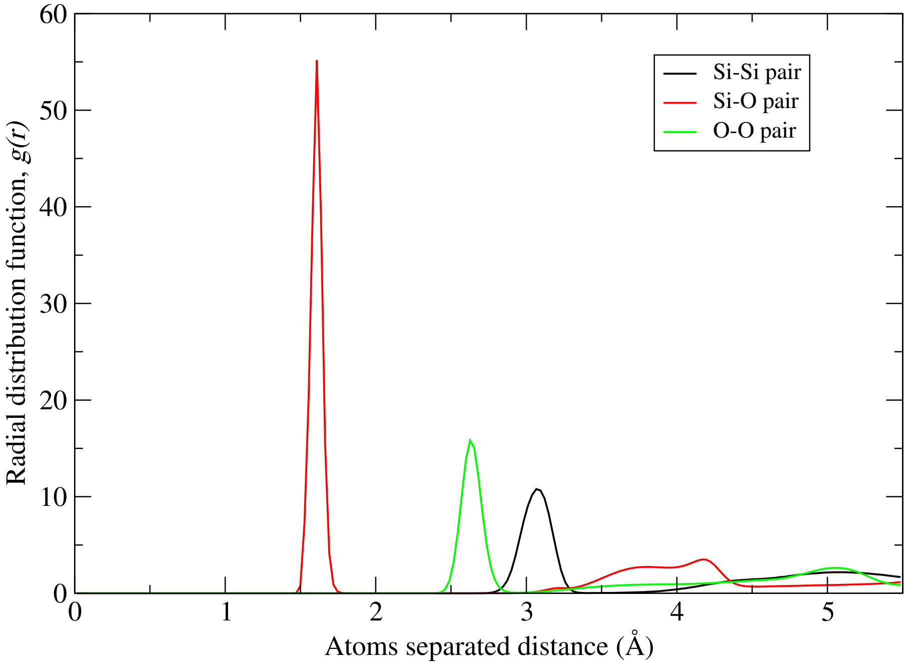

Figure S1. Radial distribution function of silica aerogel.

Indentation load-depth (P-h) curves of nanocomposites with silica aerogel slab of type I

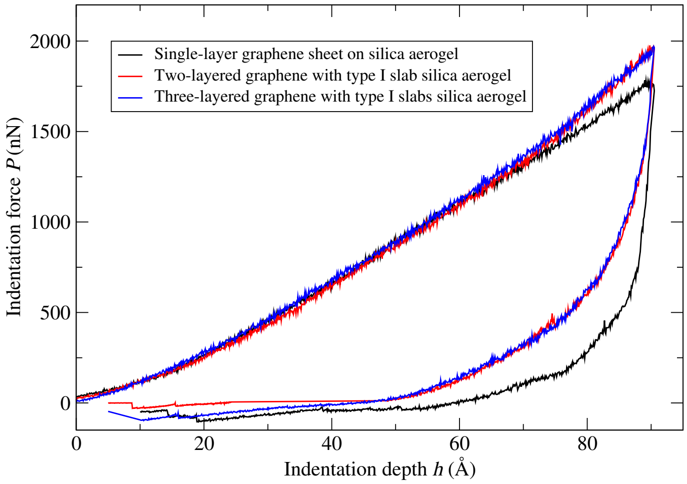

Figure S2. Indentation force−depth ($P-h$) curves of the two-layered and three-layered graphene-reinforced with silica aerogel of slab type I nanocomposites. As the number of the graphene layers increase the plastic deformation energy decreases significantly.

Indentation load-depth (P-h) curves of nanocomposites with silica aerogel slab of type II

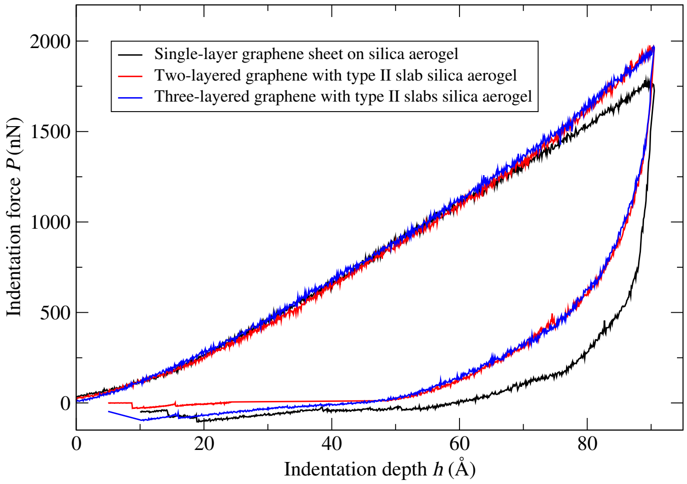

Figure S3. Indentation force−depth ($P-h$) curves of the two-layered and three-layered graphene-reinforced with silica aerogel of slab type II nanocomposites. As the number of the graphene layers increase the plastic deformation energy decreases significantly.

References

1. Murillo JSR, Bachlechner ME, Campo FA, Barbero EJ (2010) J Non-Cryst Solids **356**: 1325–1331.

2. Vashishta P, Kalia RK, Rino JP, Ebbsjö I (1990) Phys Rev B **41**: 12197–12209.
